# Evolving Standards in Prosthetic Heart Valve Assessment With Cardiovascular Imaging: Key Changes in the 2024 American Society of Echocardiography Guidelines

**DOI:** 10.1016/j.shj.2024.100372

**Published:** 2024-11-30

**Authors:** Fraser Graham, Stephen Dobbin, Maala Sooriyakanthan, Wendy Tsang

**Affiliations:** a British Heart Foundation Cardiovascular Research Centre, University of Glasgow, Glasgow, UK; b Division of Cardiology, Toronto General Hospital, University of Toronto, Toronto, Canada

**Keywords:** Cardiac valves, Echocardiography, Imaging, Prosthetic valves

## Abstract

The American Society of Echocardiography recommendations published in 2024 for evaluating prosthetic heart valve (PHV) function with cardiovascular imaging include new recommendations for the use of cardiac magnetic resonance imaging, cardiac computed tomography, and cardiac positron emission tomography. Additionally, they now provide normative echocardiographic values for right-sided PHVs and transcatheter heart valves in native valves and when used for valve-in-valve procedures. Furthermore, the recommendations include definitions to improve the recognition and classification of prosthetic heart valve dysfunction. The aim of this review is to summarize these key changes compared to the 2009 update and to include useful tables and figures to aid the reader in assessment of PHV function.

## Highlights

• Reviews new recommendations for the use of complimentary imaging modalities.
• Discusses the updated definitions for structural valve deterioration.
• Summarizes new echo criteria and thresholds for prosthetic heart valve (PHV) stenosis and regurgitation.
• Emphasizes core practices when assessing PHVs.
• Highlights expanded normal echo valves for contemporary PHVs in all positions.

## Graphical abstract

**Figure:**
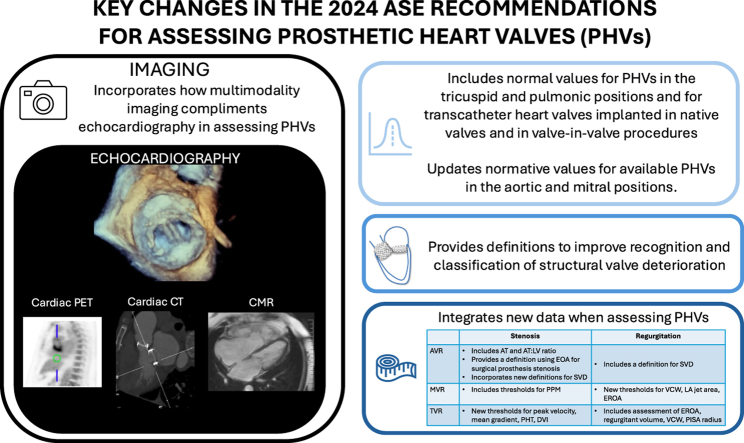

## Introduction

The American Society of Echocardiography (ASE) recommendations for the assessment of prosthetic heart valves (PHVs) with cardiovascular imaging were last updated in 2009.^1^ During the intervening 15 years, there have been many changes. First, there has been the development of transcatheter heart valves (THVs) to treat native valve diseases. These THVs have also been used for valve-in-valve (ViV) and valve-in-ring procedures to address failing bioprosthetic or repaired valves. With widespread adoption of these THV interventions, normative echocardiographic values are now available. Furthermore, work from the Valve Academic Research Consortium has resulted in definitions that improve the recognition and classification of bioprosthetic heart valve dysfunction. Finally, cardiac imaging modalities such as three-dimensional (3D) echocardiography, cardiac magnetic resonance imaging (CMR), cardiac computed tomography (CCT), and cardiac positron emission tomography (PET) have become widely available and integrated into clinical practices. These imaging modalities have improved our assessment of PHVs, addressing important diagnostic questions pertaining to PHV dysfunction severity and mechanism.

The aim of this review is to summarize key changes in the updated ASE recommendations for evaluating prosthetic valves with cardiovascular imaging compared to the 2009 update.^2^ We will review central concepts in assessing PHVs, the role of echocardiography and multimodality imaging, and changes in assessing PHVs in specific cardiac positions. It must be noted that these PHV recommendations are meant to be complimentary to the ASE publication in 2019 for assessing regurgitation related to percutaneous valve repair or replacement.^3^

## General Considerations

Fueled by the clinical need for therapeutic strategies to treat severe aortic stenosis in patients with prohibitive surgical risk and subsequent clinical trial successes in lower-risk patients, both balloon-expandable (e.g., SAPIEN, Edwards Lifesciences) and self-expandable transcatheter aortic valve implantation (TAVI) valves (e.g., Evolut, Medtronic) are now commonly used.^4^, ^5^, ^6^ This success has led to the development of transcatheter pulmonary valves for failing conduits and the use of TAVI valves to treat deteriorating bioprosthetic valves and surgically repaired valves with annuloplasty rings. While surgical bioprosthetic and mechanical prostheses are still widely implanted, over the past decade, there has been a trend toward fewer mechanical valves, possibly explained by the growing use of transcatheter valves and a belief in the potential for future transcatheter interventions for failing bioprosthetic valves.^7^

In this landscape, information on the implanted valve type, position, and size remains central in these guidelines on PHV assessment. This is due to differences in the imaging characteristics as well as the hemodynamic and complication profiles between surgical mechanical, surgical bioprosthetic, and transcatheter heart valves. The echocardiographer requires this knowledge to not only recognize normal PHV function but also to diagnose and grade levels of dysfunction based on deviations from expected measurement values. The 2009 recommendations only included tables with normal echocardiographic reference values for prosthetic valves in the aortic and mitral positions. The updated recommendations provide normal reference values for common TAVI valve sizes and types implanted not only in the aortic position but also when these valves are employed as ViV or valve-in-ring implants in the aortic, mitral, tricuspid, and pulmonic valve positions. Normal values are also provided for surgical bioprosthetic and mechanical PHVs in the tricuspid and pulmonic positions, and for pulmonary homografts and valved conduits.

## PHV Imaging

### Echocardiography

**Figure 1:**
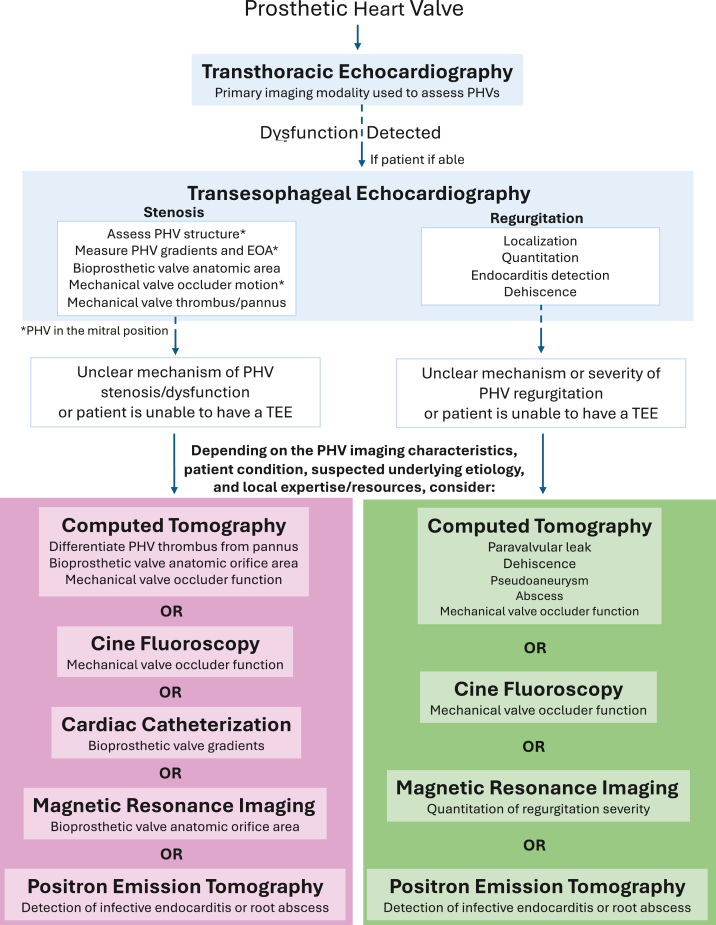
Flow chart of imaging modalities that can be used to assess prosthetic heart valve stenosis and regurgitation with a focus on the major strengths of each modality. Abbreviations: EOA, effective orifice area; TEE, transesophageal echocardiography; PHV, prosthetic heart valve.

While the role of multimodality cardiac imaging is expanded in these new recommendations, transthoracic echocardiography (TTE) remains the primary imaging modality for the initial and routine assessment of PHVs (Figure 1). High-quality echocardiographic imaging that allows comparison of serial measurements remains an important principle in these updated guidelines. In general, fundamental 2D and Doppler echocardiographic concepts that impact PHV assessment, such as the pressure recovery phenomenon, are largely unchanged from 2009. Most decision-making on the presence and severity of PHV dysfunction is based on a combination of 2D echocardiographic appearances and Doppler-derived measurements. However, as will be discussed in the aortic PHV subsection, there have been changes in how stenotic lesions are diagnosed and graded for PHV in the aortic position and the criteria used to assess prosthesis-patient mismatch.

These updated guidelines also emphasize the importance of integrating both flow-dependent and less flow-dependent echocardiographic parameters when assessing PHVs for stenosis, especially in the aortic position. Flow-dependent parameters such as peak and mean gradients are subject to changes independent of valvular function due to alterations in heart rate and hemodynamic status. Integrating less flow-dependent parameters such as PHV orifice areas in stenosis helps avoid potential misdiagnosis from using flow-dependent measurements alone.

**Figure 2:**
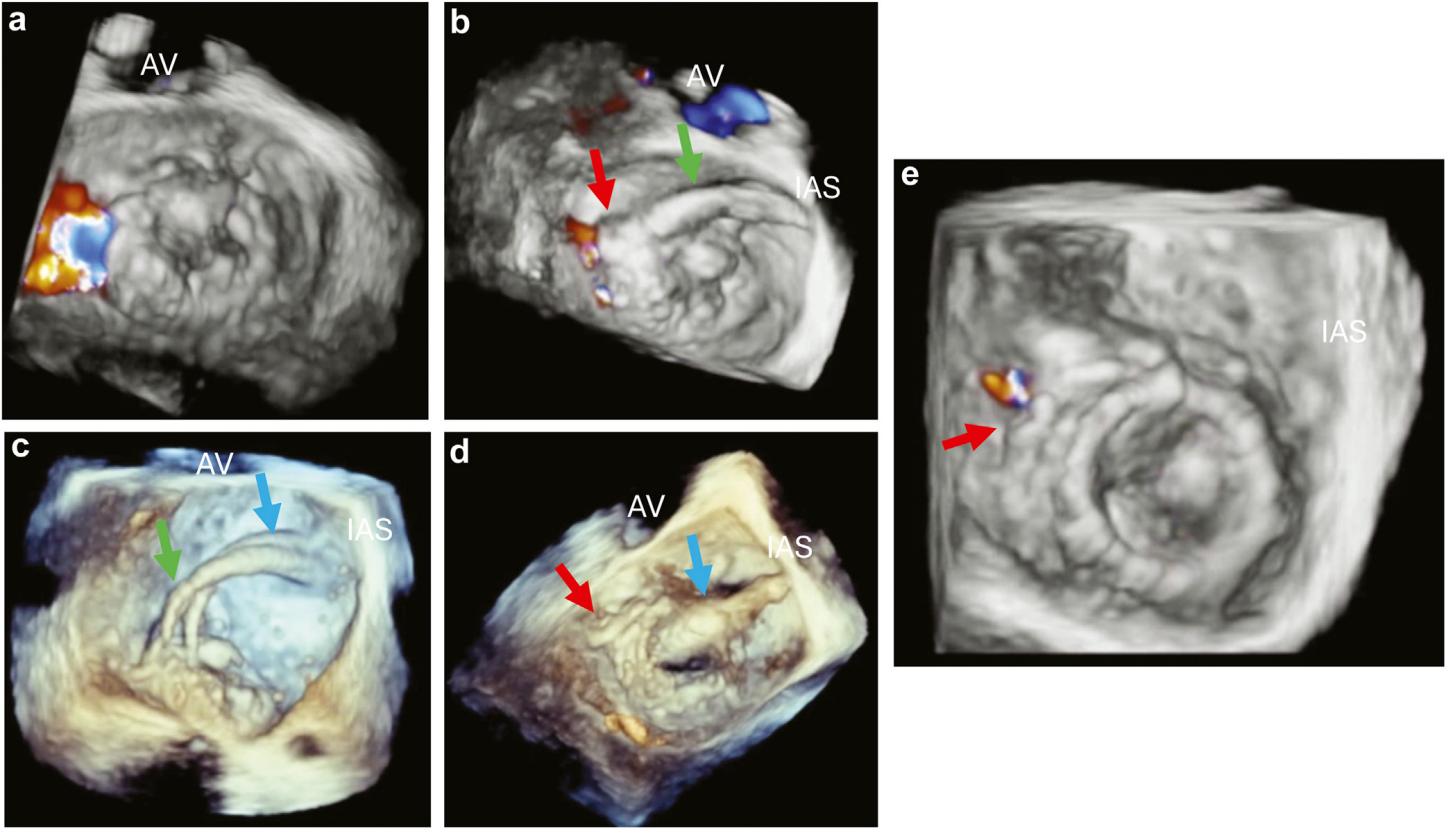
**Patient with a bioprosthetic heart valve in the mitral position with severe valvular stenosis and severe paravalvular regurgitation**. (a) Three-dimensional transesophageal echocardiographic *en face* view of the bioprosthetic valve in the mitral position during systole with severe paravalvular regurgitation located laterally. (b) Postplacement of a paravalvular leak closure device (red arrow). It is still attached to the delivery catheter (green arrow), which can be seen crossing the interatrial septum. Residual trace paravalvular regurgitation is still present. (c) Placement of the delivery catheter (blue arrow) across the prosthetic heart valve in preparation for the valve-in-valve procedure. (d) Postballoon inflation of the transcatheter aortic valve implantation (TAVI) valve within the bioprosthetic valve. The balloon (blue arrow) is still present across the TAVI valve-in-valve. The paravalvular leak closure device is also visualized (red arrow), but it is no longer attached to the delivery catheter. (e) Three-dimensional transesophageal echocardiographic *en face* image post-TAVI valve-in-valve demonstrating trivial paravalvular regurgitation from adjacent to the paravalvular closure device (red arrow). Abbreviations: AV, aortic valve; IAS, interatrial septum.

Transesophageal echocardiography (TEE) remains a mainstay in the evaluation of dysfunctional PHVs and for preprocedural assessments. Postintervention, it is vital in assessing PHV function and for complications, including paravalvular regurgitation. The updated guidelines highlight the value of 3D TEE, particularly for PHVs in the mitral position, where the *en*-*face* view allows complete visualization of the PHV in a single image (Figure 2). Another addition to the updated recommendations is in the use of 2D and 3D TEE for guiding mitral and tricuspid transcatheter valve replacements and in treating PHV paravalvular regurgitation. The new recommendations also briefly outline helpful TEE views for transcatheter tricuspid valve procedures, including the deep esophageal inflow-outflow view located between 40 and 60° with the orthogonal 140° bi-plane view. Intracardiac echocardiography is included in this update for its role in transcatheter pulmonic valve replacement and potential role in tricuspid valve procedures.

### Role of Multimodality Imaging

More guidance has been provided on the use of multimodality imaging, specifically on how and when to use cross-sectional (CCT and CMR) and metabolic tracer (cardiac PET) imaging (Table 1). This has been driven by both widespread clinical experience and research performed during the intervening years between guidelines.

**Table 1:** Imaging strengths and limitations in PHV assessment∗

	Imaging strengths	Imaging limitations	Valves
TEE	• Portable • High spatial and temporal resolution in real-time allow detection of small mobile masses and jets • Provides Doppler quantitative assessment • 3D en face views provide complete PHV visualization in a single image • MPR analysis allows more definitive assessments	• Image quality depends on imaging windows and valve orientation • Imaging artifacts from the PHV that may shadow the farfield or other PHVs	• PHV in the mitral, then aortic, tricuspid, and pulmonic positions
ICE	• Superior 2D and 3D en face visualization of PHVS located anteriorly (pulmonary or tricuspid position)	• Limited 3D pyramid size • Limited 3D temporal and spatial resolution with and without color	• PHV in the pulmonic and tricuspid position
CCT	• Good spatial resolution • Image quality is not limited by PHV position or the presence of multiple PHVs	• Unable to provide hemodynamic information • Small regurgitant jets can be missed as regurgitation severity is based on anatomic defects • Beam-hardening artifacts from mechanical PHVs may interfere with assessment • Nephrotoxic agents are needed for CT angiography • Complete cardiac cycle acquisition increases radiation exposure • Limited temporal resolution	• PHV in all valve locations
CMR	• Able to quantitate bioprosthetic peak velocities and gradients for all valve locations • Able to quantitate regurgitant volumes and fraction	• Limited spatial and temporal resolution • Artifacts from mechanical PHV and some bioprosthetic valves limit assessments • Irregular heart rhythms limit PHV visualization and flow quantitation • Contraindicated in patients with cardiac implantable electronic devices, cochlear implants, ferromagnetic implants/devices (i.e., aneurysm clips), and neurostimulators • Challenging for patients with claustrophobia or who are clinically unstable	• PHV in all valve locations

Abbreviations: 3D, three-dimensional; CCT, cardiac computed tomography; CMR, cardiac magnetic resonance imaging; CT, computed tomography; ICE, intracardiac echocardiography; TEE, transesophageal echocardiography; PHV, prosthetic heart valve.

∗ Edited from Zoghbi et al.^1^

**Figure 3:**
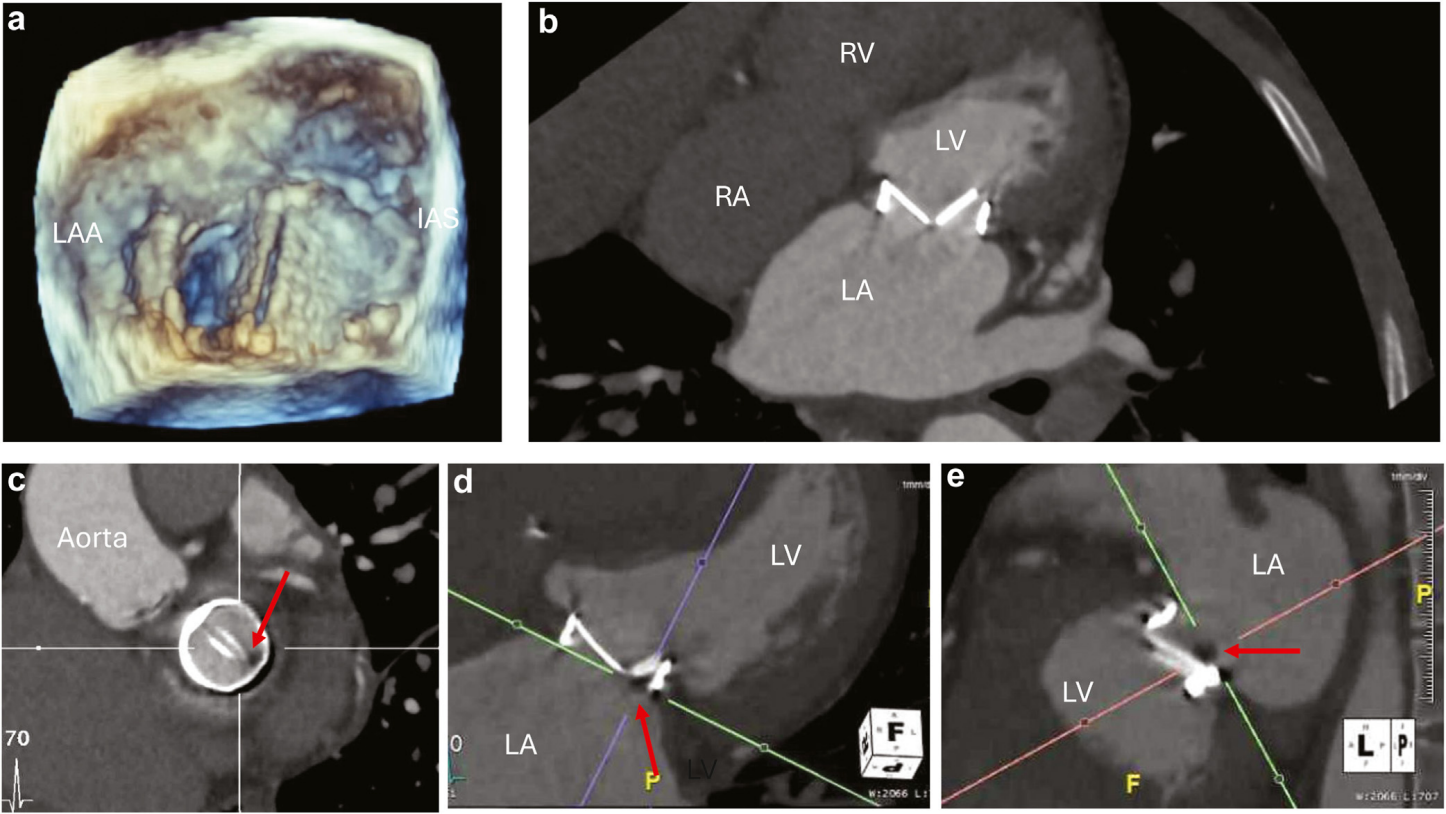
**Patient with a mechanical valve in the mitral position with stenosis from a fixed medial occluder secondary to thrombus**. (a) Three-dimensional (3D) transesophageal echocardiographic *en*-*face* view of a mechanical valve in the mitral position during diastole with a fixed medial occluder. The lateral occluder opens normally. (b) Cardiac computed tomography with contrast demonstrates the same findings. (c to e) Cardiac computed tomography multiplanar reconstructed views demonstrate the presence of a mass (red arrow) on the atrial side at the junction of the valve discs. The mass measured up to 5 mm and had low attenuation of 80-90 Hounsfield units, which is consistent with thrombus. Abbreviations: LA, left atrium; LAA, left atrial appendage; LV, left ventricle; RA, right atrium; RV, right ventricle.

Improvements in scanner capabilities and the advent of electrocardiographic gating have greatly enhanced the utility of CCT in assessing both bioprosthetic and mechanical PHVs (Figure 3). Its high spatial resolution is particularly beneficial when dysfunction detected by echocardiography has an unclear etiology or when structural intervention is being considered. For patients with echocardiographic findings of PHV stenosis, CCT, in addition to TEE, provides valuable information on bioprosthetic anatomic orifice area and mechanical PHV occluder motion. Using contrast, CCT is also effective in differentiating between stenosis caused by thrombus and that caused by pannus formation. In cases of PHV regurgitation, CCT compliments TEE in assessing valve dehiscence. Additionally, complications of infective endocarditis, such as paravalvular leaks (PVLs) and pseudoaneurysms, can be localized by CCT, and delayed phase scanning can be particularly helpful in identifying abscess cavities.

All PHVs can be safely imaged by 1.5 or 3T magnetic resonance imaging scanners.^8^^,^^9^ In cases where PHV regurgitation has been identified but echocardiographic findings are discrepant, CMR can improve grading of regurgitation severity. The guidelines highlight that CMR is particularly valuable for this purpose in patients with PHVs. CMR methods, such as through-plane phased contrast imaging, offer accurate quantification of regurgitation in PVL cases without relying on accurate 2D measurements of aortic valve annular or semilunar valve outflow tract dimensions, as required in echocardiography.

**Figure 4:**
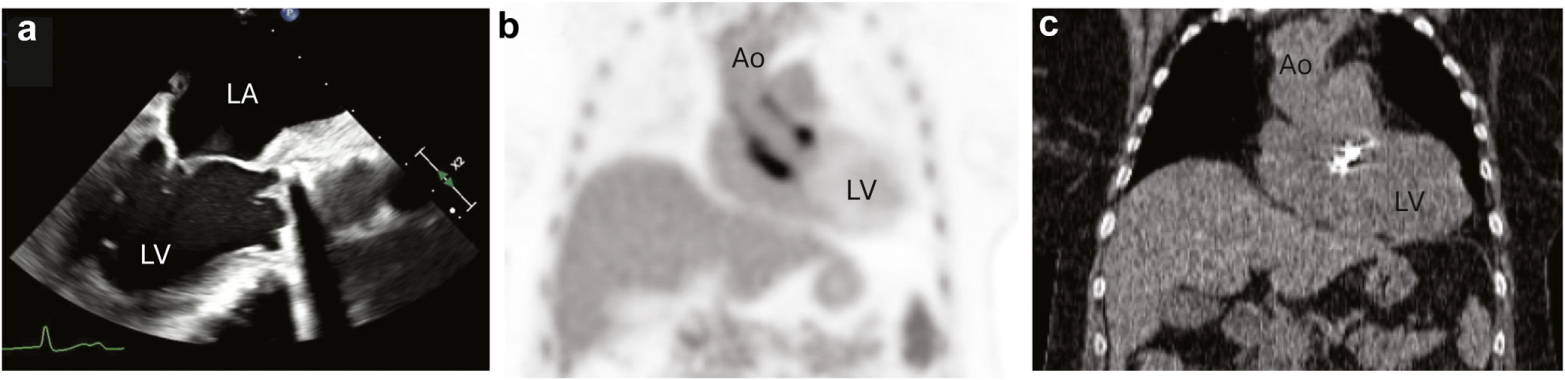
**Patient with infective endocarditis of post**-**Bentall with a mechanical valve**. (a) Transesophageal echocardiographic image during diastole demonstrating vegetations on the mechanical valve in the aortic position and a thickened aortic root and ascending aorta. (b) Fludeoxyglucose F18 positron emission tomography (FDG-PET) demonstrating activity involving the mechanical aortic valve and aortic root. (c) The matching cardiac computed tomography image demonstrating the location of the mechanical aortic valve. Abbreviations: Ao, aorta; LA, left atrium; LV, left ventricle.

The predominant role of cardiac PET, typically with flurodeoxyglucose, in PHVs is in the assessment of infective endocarditis and/or root abscess (Figure 4).^10^^,^^11^ Its use should be complimentary to both echocardiography and computed tomography (CT), but caution is needed as tracer uptake in the aortic root can be a normal phenomenon up to 1 year postsurgery.^12^^,^^13^

While the roles for echocardiography, CMR, CCT, and cardiac PET in assessing PHV are specifically delineated in the current guidelines, other modalities such as intracardiac echocardiography, stress echocardiography, cinefluoroscopy, and cardiac catheterization also provide valuable insights. The choice of imaging modality will depend on the type and position of the PHV, the strengths and limitations of each modality, the local availability and expertise, the patient’s clinical condition, and the suspected underlying etiology of the PHV stenosis or regurgitation.

## Classification of Prosthetic Heart Valve Dysfunction

**Figure 5:**
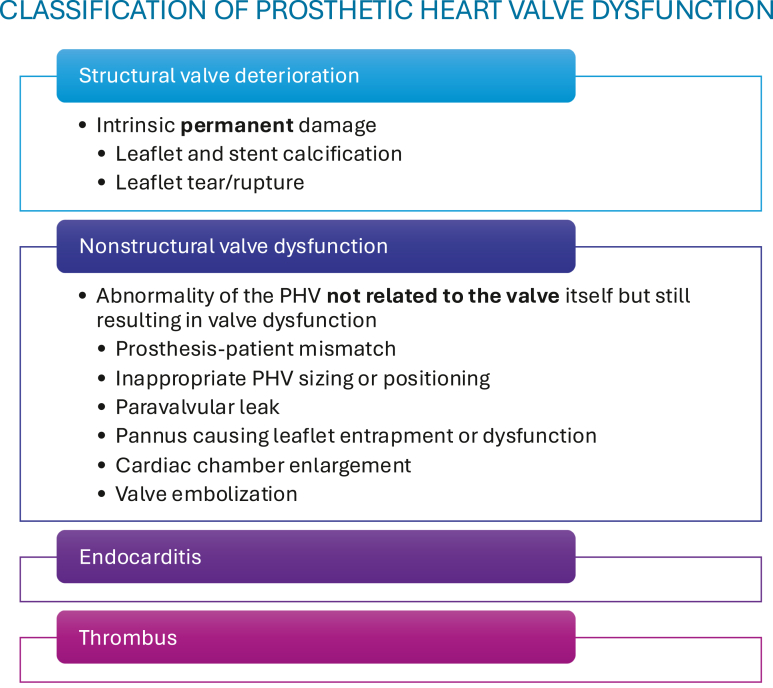
Classification scheme for prosthetic valve dysfunction. Abbreviation: PHV, prosthetic heart valve.

The 2024 ASE PHV recommendations have further refined the classification of heart valve dysfunction based on work from the Valve Academic Research Consortium 3. Four categories of PHV dysfunction are described in the new recommendations (Figure 5). These include structural valve deterioration (SVD), nonstructural valve dysfunction, endocarditis, and thrombus. SVD is defined by intrinsic, irreversible, or permanent PHV damage such as leaflet “wear and tear,” calcification and fibrosis, leaflet disruption, and stent or strut fracture or deformation.^2^ Most of these pathological changes occur toward the end of a PHV’s lifespan. Nonstructural valve dysfunction is defined as “any abnormality of the prosthesis not related to the valve itself but still resulting in valve dysfunction.” This includes prosthesis-patient mismatch, PVLs, and pannus ingrowth causing leaflet entrapment. Endocarditis remains an important challenge in PHVs with a prevalence of 1% to 6%.^14^^,^^15^ Knowledge of typical echocardiographic features of endocarditis in PHVs including complications such as PVL, abscess, and pseudoaneurysm is imperative to facilitate early diagnosis and appropriate treatment. Thrombus can occur on mechanical or bioprosthetic valves and are more common in right-sided than left-sided valves. Typical appearances of a homogenous, usually immobile, echogenic mass are not always present, and use of cardiac CT, particularly in bioprosthetic and TAVI valves where the only feature may be leaflet thickening and increased gradients, can improve discrimination from pannus.

## Evaluation of Prosthetic Valves

### Aortic

The guidance on integrated assessment of PHVs in the aortic position, predominantly using Doppler echocardiography, is largely unchanged. Main updates include small, but important, changes to the algorithmic assessment of high peak velocities through prosthetic aortic valves, specific considerations for surgical aortic valve replacements, guidance on serial assessments of TAVI valves, and normal values for TAVI valves for both native and ViV procedures.

#### Evaluation of Stenosis

**Figure 6:**
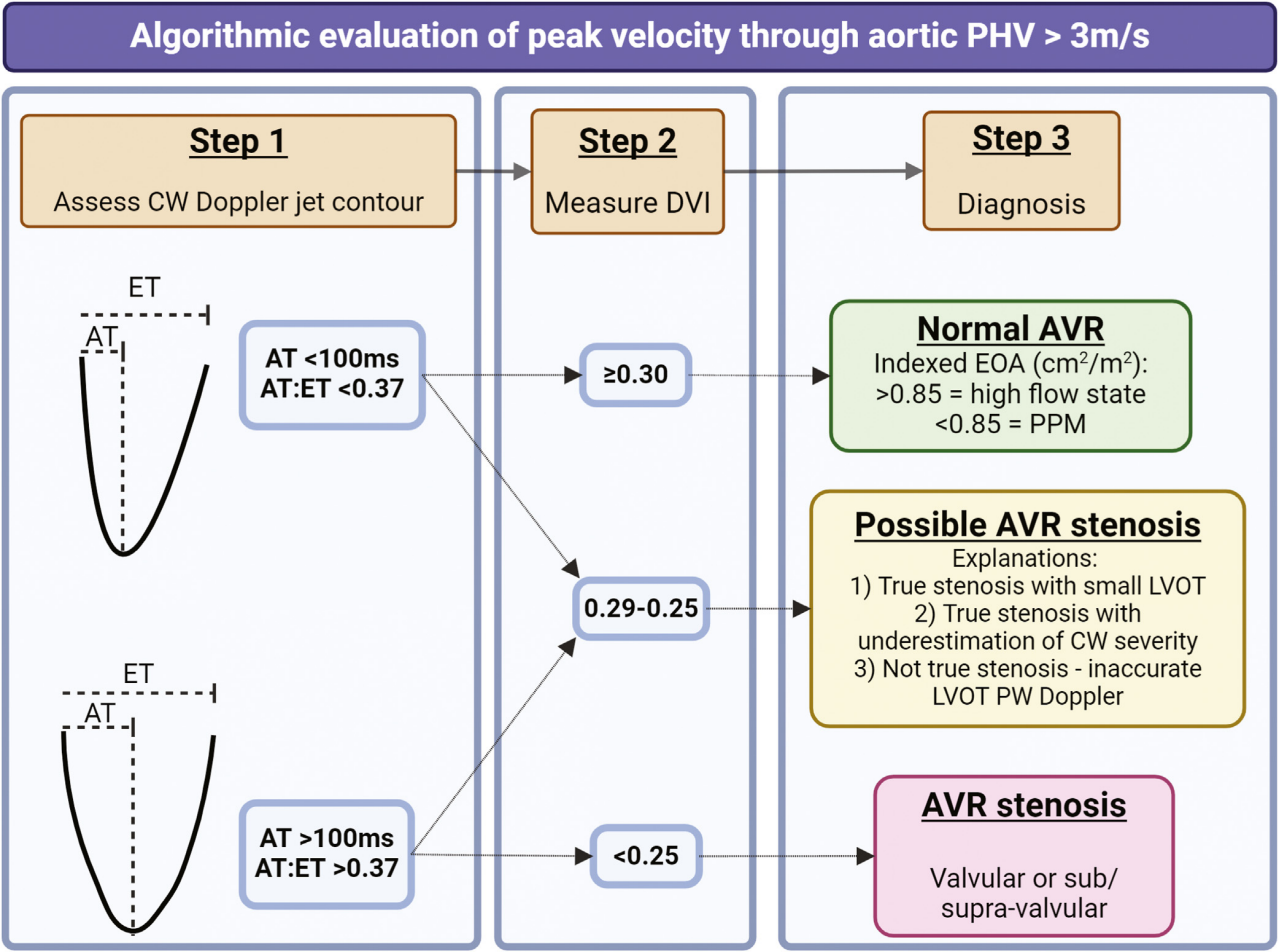
**Flow chart detailing algorithmic evaluation of possible aortic PHV stenosis**. Edited from Zoghbi et al.^1^ Abbreviations: AT, acceleration time; AVR, aortic valve replacement; CW, continuous wave; DVI, dimensionless valve index; EOA, effective orifice area; ET, ejection time; LVOT, left ventricular outflow tract; PHV, prosthetic heart valve; PPM, patient-prosthesis mismatch; PW Doppler, pulsed-wave Doppler.

In the presence of elevated peak velocities (>3 m/s), an updated diagnostic algorithm encouraging earlier use of acceleration time and incorporating the acceleration time: ejection time ratio is provided (Table 2). Although not new, the value of these parameters has gained recognition in recent years in terms of helping to determine causes of raised PHV gradient.^16^ Together with accurate left ventricular outflow tract pulsed-wave Doppler and calculation of Doppler velocity index (DVI), these Doppler-derived parameters can differentiate stenosis from other causes in most cases. Importantly, incorporation with imaging visualizing leaflet structure and mobility via TTE, TEE, and/or CCT is paramount. Leaflets or occluders that are obviously thickened or obstructed with high PHV gradients are generally diagnostic of true stenosis, whereas normal leaflet/occlude function is suggestive of other causes such as high-flow states or prosthesis-patient mismatch (PPM). It is emphasized that significant stenosis should meet at least one flow-dependent and one flow-independent criteria. An edited version of the algorithm provided in the updated guideline is presented in Figure 6.

**Table 2:** Summary of changes for replaced aortic valves

Aortic valve replacement
**Stenosis** • Incorporates acceleration time into the list of Doppler parameters to consider when assessing PHV in the aortic position and acceleration time to ejection time ratio in assessing elevated PHV velocities • For surgical PHVs, specifies that a calculated EOA, i.e., more than 2 SDs smaller than the reference EOA, is suggestive of significant stenosis. • Includes definition of PPM with an indexed EOA of ≤0.65 cm2 (body mass index [BMI] <30 kg/m^2^) or ≤0.55 cm^2^ (BMI ≥30 kg/m^2^) diagnostic of severe PPM provided they are not within 1 SD of the reference EOA for that valve size and type. • Includes a definition for significant SVD, which is an increase in mean gradient (MG) ≥20 mmHg to a MG of ≥ 30 mmHg with paired decrease in EOA ≥0.6 cm^2^ or ≥50% and/or a decrease in DVI ≥0.2 or ≥40% compared to baseline postprocedural study. A MG change of ≥10 mmHg with a concomitant decrease in EOA ≥0.3 cm^2^ and/or decrease in DVI >0.1 or >20% compared with baseline is suggestive of possible SVD.

	Significant Stenosis
Acceleration time	>100 msec
Acceleration time: LV ejection time ratio	>0.37
**Regurgitation** • Criteria for grading AR severity are unchanged. • Quantitative parameters should be used when possible. • Inclusion of a definition of significant SVD, which is a new occurrence or increase of at least 2 grades of intraprosthetic AR to moderate or greater to severe AR. A new occurrence or increase of at least one grade of intraprosthetic AR to moderate or greater AR is suggestive of possible SVD.	

Abbreviations: AR, aortic regurgitation; DVI, Doppler velocity index; EOA, effective orifice area; LV, left ventricular; PHV, prosthetic heart valve; PPM, patient-prosthesis mismatch; SVD, structural valve deterioration.

The table delineating normal from possible or significant stenosis using Doppler parameters has also been changed to include specific considerations for surgical and TAVI valves. For surgical aortic valve replacements, a mean gradient of greater than 35 mmHg still suggests significant stenosis. Updated normal values for PHV in the aortic position include effective orifice areas (EOA) for most implanted valves. This information is needed to determine if the calculated EOA differs from the reference EOA by 1 or 2 SDs. For surgical aortic valve replacements, a calculated EOA that is more than 2 SDs smaller than the reference EOA suggests significant stenosis. Specific EOA thresholds, indexed to body mass, are also important in identifying PPM with an indexed EOA of ≤0.65 cm^2^ (BMI <30 kg/m^2^) or ≤0.55 cm^2^ (BMI ≥30 kg/m^2^) diagnostic of severe PPM, provided they are not within 1 standard deviation of the reference EOA for that valve size and type.

The new recommendation also provides new criteria to identify SVD in the aortic position for both stenosis and regurgitation. In terms of stenosis, increases in mean gradient (MG) ≥20 mmHg to a MG ≥ 30 mmHg with paired decrease in EOA ≥0.6 cm^2^ or ≥50% and/or a decrease in DVI ≥0.2 or ≥40% compared to the baseline postprocedural study are considered to identify significant SVD. The definition for regurgitation is provided in the following section.

#### Evaluation of Regurgitation

Specific mention has been made in the new guidelines of how to practically assess for the location and mechanism of regurgitation associated with aortic PHVs, namely by sweeping in both parasternal long- and short-axes views. Similar to stenosis, new hemodynamic criteria to define SVD with respect to regurgitation have been suggested: new occurrence or increase of at least 2 grades of intraprosthetic aortic regurgitation (AR) to moderate or greater is considered significant SVD. New occurrence or one grade increase of intraprosthetic AR to moderate or greater is considered a possible SVD.

**Figure 7:**
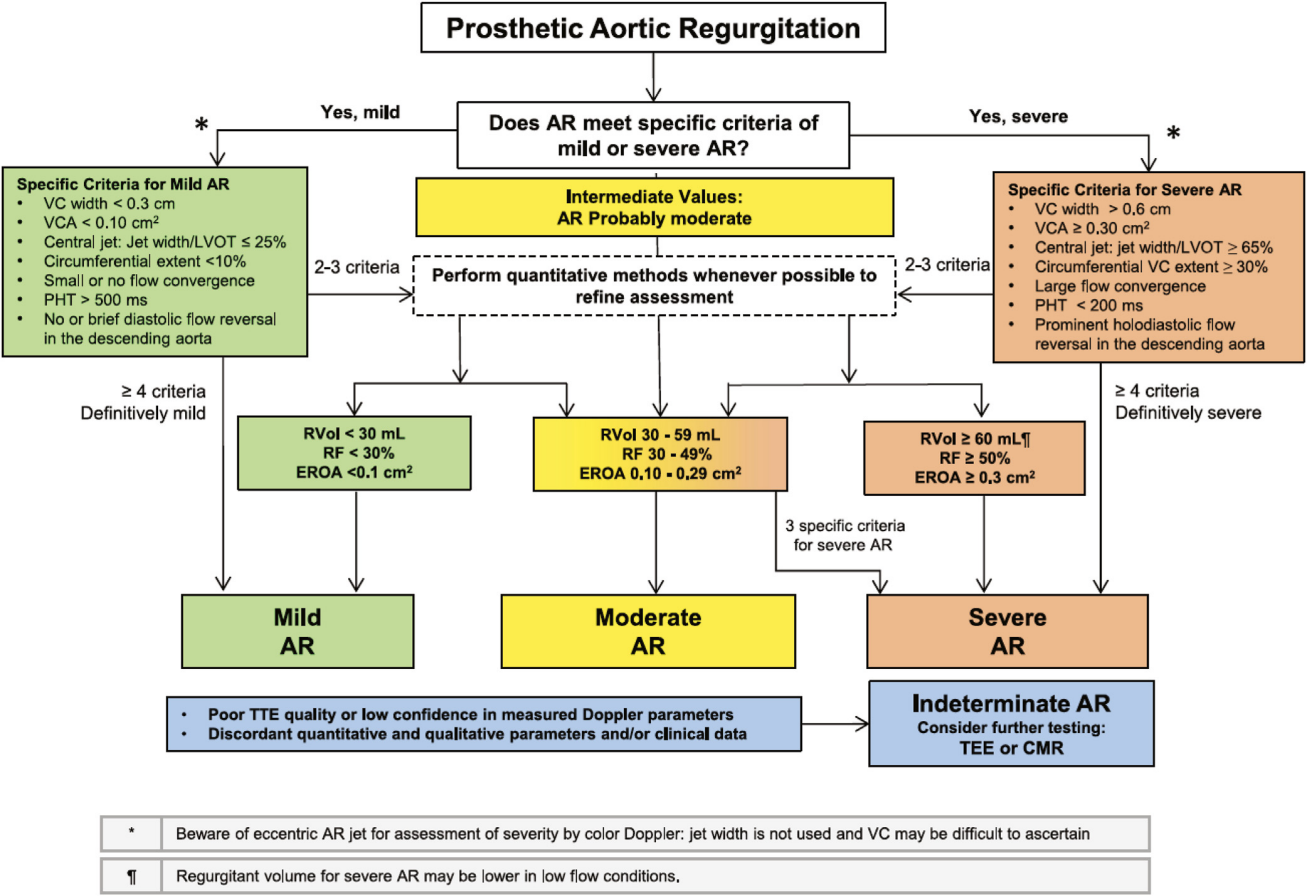
**Algorithm using echocardiographic parameters to classify regurgitation severity for prosthetic heart valves in the aortic position**.**∗Reprinted from the Journal of the American Society of Echocardiography, 37/1, William A. Zoghbi, Pei-Ni Jone, Mohammed A. ChamsiPasha, Tiffany Chen, Keith A. Collins, Milind Y. Desai, Paul Grayburn, Daniel W. Groves, Rebecca T. Hahn, Stephen H. Little, Eric Kruse, Danita Sanborn, Sangeeta B. Shah, Lissa Sugeng, Madhav Swaminathan et al., Guidelines for the Evaluation of Prosthetic Valve Function With Cardiovascular Imaging: A Report From the American Society of Echocardiography Developed in Collaboration With the Society for Cardiovascular Magnetic Resonance and the Society of Cardiovascular Computed Tomography, 2-63, Copyright (2024), with permission from Elsevier.** Abbreviations: AR, aortic regurgitation; CMR, cardiac magnetic resonance imaging; EROA, effective regurgitant orifice area; LVOT, left ventricular outflow tract; PHT, pressure half-time; RF, regurgitant fraction; Rvol, regurgitant volume; TEE, transesophageal echocardiography; TTE, transthoracic echocardiography; VC, vena contracta; VCA vena contracta area.

Doppler-based grading of AR severity is similar to those suggested in native valves and to guidance from 2009.^2^ A new helpful algorithm (Figure 7)^1^ outlines the use of qualitative, quantitative, and semiquantitative measures to define severity. Quantitative measures are advised wherever possible. At least 4 criteria should be met to determine severity.

#### Evaluation of TAVI Valves

Assessment of TAVI valve function should include both flow-dependent (peak and mean gradients) and less flow-dependent (EOA) parameters. Velocity and velocity time integral (VTI) will be higher when sampled within the stents of transcatheter valves. It is stressed in the guidelines that measurement of left ventricular outflow tract diameter should mainly remain “outer-edge to outer-edge” in annular implants, that is immediately proximal to the stent struts. The corresponding VTI should be measured in the same position to avoid capturing flow acceleration at the inlet of the stent struts. The exception to this is in low-lying self-expandable valves where the “inner-edge to inner-edge” left ventricular outflow tract (LVOT) diameter should be taken within the lower end of the stent struts but before the flow acceleration at the level of the valve cusps, with VTI measurements in the same position.

One specific change in these guidelines is an emphasis on the role of serial Doppler measurements in TAVI valves and how to relate the degree of change from baseline to possible or significant TAVI valve stenosis/SVD. The criteria used are identical to those described above for stenosis-related SVD (Table 2). Finally, the updated recommendations provide normal values for the most common TAVI valves of various sizes including balloon expandable valves utilized as ViV implants, which aid in this assessment.

Retrospective electrocardiogram-gated assessment by CCT can be particularly helpful in diagnosing leaflet thrombosis in TAVI valves. It is also fundamental in assessing risk of coronary ostia occlusion in ViV procedures in the aortic position and in assessing risk of left ventricular outflow tract obstruction and annular sizing in cases of ViV in the mitral position, typically post-SVD following mitral repair or bioprosthetic replacement.^16^^,^^17^

### Mitral

The recommendations for the evaluation of PHVs in the mitral position similarly focus on the importance of Doppler echocardiography via both TTE and TEE. The main updates include reference ranges for transcatheter ViV prosthetic valves as well as highlighting the complementary role of other imaging modalities, including CMR and CCT. Given the challenges of assessing the severity of PHV dysfunction in the mitral position using echocardiography and the increasing number of patients eligible for transcatheter ViV intervention, the use of multiple imaging modalities to aid in determining the severity and mechanisms of PHV dysfunction in the mitral position is becoming ever more important.

#### Evaluation of Stenosis

Doppler echocardiography remains the mainstay of evaluation of prosthetic mitral stenosis, and the diagnostic criteria for significant prosthetic mitral stenosis are essentially unchanged from 2009. These include the measurement of the mean gradient at a normal heart rate, pressure half-time (PHT), DVI, and EOA. It is proposed that CMR may play a role in assessing leaflet mobility, direct planimetry of bioprosthetic valves, and measuring peak velocity through the prosthetic valve via the use of phase-contrast imaging sequences. All of these may be limited by artifact, particularly with mechanical valves, and given the limited validation of these methods, the role of CMR remains complementary at this stage. Similarly, it is suggested that CCT may play a role in determining the mechanism of stenosis, particularly in the context of pannus. Additionally, multiplanar imaging allows for accurate measurement of the geometric orifice area. TEE also allows for *en face* imaging of the mitral valve, facilitating assessment of the etiology of stenosis and, via 3D multiplanar reconstructions, mitral valve area. PPM may also occur in the mitral position but is much less common than in the aortic position with severe PPM being defined as an EOA ≤0.90 cm^2^ (BMI <30 kg/m^2^) or ≤0.75 cm^2^ (BMI ≥30 kg/m^2^) in apparently normal functioning valves (Table 3).

**Table 3:** Summary of changes for assessing replaced mitral valves

Mitral valve replacement
**Stenosis** • Thresholds for stenosis assessment are unchanged. • Thresholds for PPM are included. Severe PPM is defined as an EOA ≤0.90 cm2 (BMI<30 kg/m2) or ≤0.75 cm2 (BMI ≥30 kg/m2). • CMR may play a role in assessing PHV structure and function. • CCT may help differentiate thrombus from pannus.
**Regurgitation** • Thresholds of MR LA jet area, vena contracta width, and EROA for severe regurgitation have changed but are consistent with the 2017 ASE native valve regurgitation cutoff values. • Includes CMR regurgitation volume via comparison of left ventricular and aortic valve stroke volumes or aortic and pulmonary valve stroke volumes • CCT may be used to measure the EROA.

	2009	2024
MR LA jet area	>40%	>50%
Vena contracta width	≥6 mm	≥7 mm or >8 mm from biplane assessment
EROA	≥0.5 cm^2^	>0.4 cm^2^
Regurgitant volume	>60 mL	>60 mL

Abbreviations: CCT, cardiac computed tomography; CMR, cardiac magnetic resonance imaging; EOA, effective orifice area; EROA, effective regurgitant orifice area; LA, left atrium; MR, mitral regurgitation; PHV, prosthetic heart valve; PPM, prosthesis-patient mismatch.

#### Evaluation of Regurgitation

**Figure 8:**
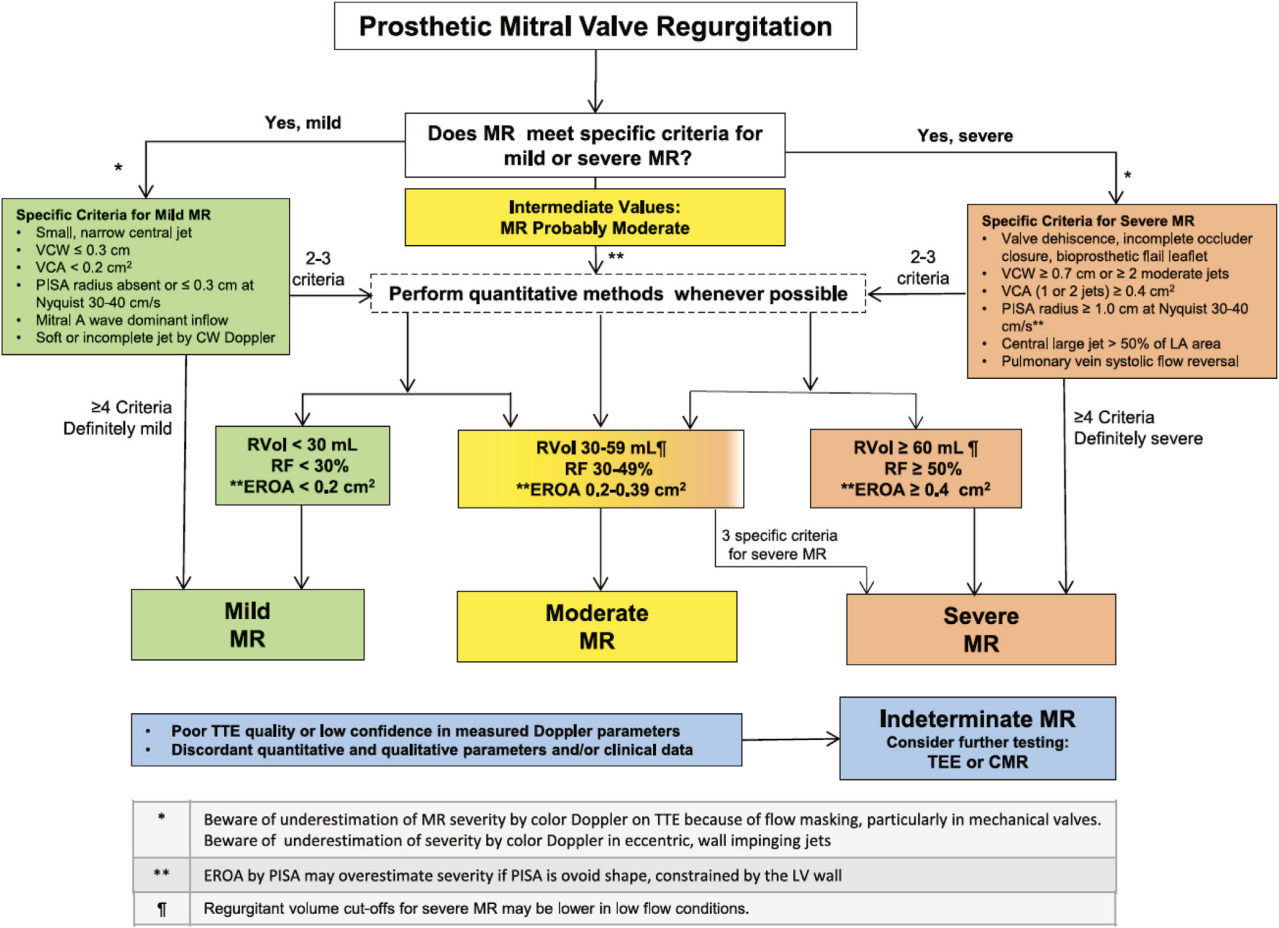
Algorithm using echocardiographic parameters to classify regurgitation severity for prosthetic heart valves in the mitral position. Reprinted with permission from the Journal of the American Society of Echocardiography . Abbreviations: CMR, cardiac magnetic resonance imaging; CW, continuous wave; EROA, effective regurgitant orifice area; LA, left atrium; LV, left ventricle; MR, mitral regurgitation; PISA, proximal isovelocity surface area; RF, regurgitant fraction; Rvol, regurgitant volume; TEE, transesophageal echocardiography; TTE, transthoracic echocardiography; VCA, vena contracta area; VCW, vena contracta width.

As TTE assessment of prosthetic mitral regurgitation (MR) is often challenging due to acoustic artifacts from the prosthesis, determining the significance of MR is reliant on indirect measures. These indirect criteria suggesting significant MR by Doppler echocardiography are similar to those recommended previously.^2^ The importance of TEE is highlighted, particularly in mechanical valves, often obscured by reverberations with TTE, particularly in the apical window. There are some minor amendments to the grading of severity of MR using combined TTE and TEE measures compared to the 2009 guidelines (Table 3) but are in concordance with the 2017 ASE recommendations for native valve regurgitation.^18^ Within Doppler parameters, the latest guidance suggests a large central color jet area of >8 cm^2^, or at least 50% of the left atrial area (previously 40%), indicates severe MR. Additionally, within quantitative parameters, a vena contracta of ≥0.7 cm (>0.8 cm for biplane) and an effective regurgitant orifice area of ≥0.4 cm^2^ are now necessary to meet criteria for severe prosthetic MR (previously 0.6 cm and 0.5 cm^2^, respectively). With the broad range of parameters used, an algorithm is provided to aid in grading and decision-making with prosthetic MR (Figure 8).

CCT and CMR again have complementary roles in assessing the degree and mechanism of prosthetic MR. CMR is particularly useful in quantitative assessment by indirect measurement of regurgitant volume and fraction via comparison of left ventricular and aortic valve stroke volumes or aortic and pulmonary valve stroke volumes. CCT may be used to measure the regurgitant orifice area, assess occluder motion, and assess potential thrombus or complications of endocarditis. All above modalities are useful in grading levels of regurgitation in patients with transcatheter edge-to-edge repair, but this is comprehensively covered in a separate guideline recommendation.^3^

### Tricuspid

The majority of surgical intervention on the tricuspid valve (TV) remains in the form of repair in the context of concomitant left-sided valve surgery.^19^ Bioprosthetic implants are more common than mechanical, despite relatively similar rates of early and late mortality/failure due to historical concerns regarding mechanical valve thrombosis in this position.^20^ More data is available on rates of SVD of prosthetic TVs than in 2009 with an observed mean of 12 years from the primary implant to a second procedure in bioprostheses.^21^ Undesirable rates of in-patient mortality from isolated TV surgery of 10% to 13% and the success of transcatheter mitral valve repair have accelerated the investigation and use of similar devices in treatment of primarily secondary tricuspid regurgitation (TR).^22^^,^^23^ Parameters such as DVI and EOA and refined thresholds for other Doppler-based measurements for echocardiographic diagnosis of TVR stenosis are newly suggested in the updated recommendation, as are recommendations for use of multimodality imaging in certain circumstances.

#### Evaluation of Stenosis

Due to the publication of more population-based data on TV replacements, the ASE recommends that a mean gradient of <6 mmHg be considered a marker of a normal bioprosthetic TV replacement function, with a measurement of 6 to 9 mmHg likely being within acceptable limits (Table 4). It is important to bear in mind, however, that smaller valve implant sizes and high-flow states may increase mean gradients in normal functioning prostheses. A continuous wave Doppler-derived E-wave peak velocity of ≥2.1 m/s in bioprosthetics and ≥1.9 m/s in mechanical valves should alert the echocardiographer of probable stenosis. A PHT of <200 msec is felt to reflect normal bioprosthetic function early after implant in contrast to the previously suggested <230 msec in 2009 recommendation.^2^ However, as PHT is influenced by heart rate, chamber compliance, and loading conditions, it should not be used as a stand-alone parameter in gauging the presence of and degree of potential stenosis in prosthetic TV replacements.^1^

**Table 4:** Summary of changes for replaced tricuspid valves

Tricuspid valve replacement
**Stenosis** • Thresholds for the presence of tricuspid valve replacement dysfunction have changed for peak velocity, mean gradient, PHT, and DVI.

	2009	2024
Peak velocity	>1.7 m/s	>2.1 m/s for bioprosthesis
		>1.9 m/s for mechanical valves
Mean gradient	≥6 mmHg	>6 mmHg
		6-9 mmHg is acceptable for bioprosthetic valves.
		>10 mmHg post-VIV is stenotic.
Pressure half time	≥230 msec	>200 msec for bioprostheses
		>130 msec for mechanical valves
Doppler velocity index	-	>2.4-3.6 for bioprosthesis
		>2.3-2.8 for mechanical valves
• A mean gradient of >10 mmHg post-TV ViV is considered stenotic. • Normal values for mechanical and bioprosthetic valves in the tricuspid position are now provided. • CMR and CCT are recommended for determining the etiology of valve dysfunction and geometric orifice areas. Validation of CMR use is lacking.		
**Regurgitation** • Inclusion of semiquantitative parameters (PISA radius) and quantitative parameters (EROA, regurgitant volume). However, validation of these quantitative parameters is lacking.		

	2009∗	2024∗
Vena contracta width	>7 mm	≥7 mm or ≥2 moderate jets
PISA radius	-	>0.9 cm
EROA	-	≥0.4 cm^2^
Regurgitant volume	-	≥45 mL

Abbreviations: CCT, cardiac computed tomography; CMR, cardiac magnetic resonance imaging; DVI, Doppler velocity index; EROA, effective regurgitant orifice area; PHT, pressure half-time; PISA, proximal isovelocity surface area; TV, tricuspid valve; ViV, valve-in-valve.

∗ Criteria for severe regurgitation.

New to the updated recommendations is the integration of DVI (VTI prosthetic TV: VTI LVOT) and EOA (stroke volume LVOT/VTI prosthetic TV) in the assessment of TV stenosis. A summary of these suggested thresholds, including the previous suggestions in 2009, is provided in Table 4. Considering TV ViV or valve-in-ring assessment, a MG >10 mmHg should be considered stenotic.

CCT and CMR can be useful in providing evidence of valve pathology (calcification, thrombus, pannus), disk occluder function and opening angles, and geometric orifice areas. CT is particularly helpful in assessing the latter, but unique scanning protocols are required to optimize resolution of right-sided structures. Of note, geometric orifice areas are typically larger than derived EOA.

#### Evaluation of Regurgitation

Recommendations for assessment of prosthetic TV regurgitation are largely unchanged from 2009. However, there is emphasis that echocardiographers focus their attention on all three regurgitant jet components when assessing severity: flow convergence, vena contracta, and jet direction. Again, modified, off-axis views in transthoracic imaging may be necessary to fully interrogate the TV regurgitation, which is especially true for mechanical prostheses. Use of quantitative parameters is suggested but may be limited given the challenging imaging, temporal variability, intrinsic low flow hemodynamics, irregular shapes of regurgitant orifices in TV replacements, and lack of validation. An elevated DVI (VTI prosthetic TV: VTI LVOT) of ≥3.3 in the context of an increased mean gradient and normal PHT should raise suspicion of significant TR. There is a lack of data on the use of 3D vena contracta area, the interpretation of which is also hampered by limitations in temporal variability, image resolution, and unknown accuracy in nonplanar orifices. An EOA of >0.4 cm^2^, regurgitant volume >45 ml and vena contracta >0.7 cm are suggestive of severe prosthetic TR. Systolic flow reversal in hepatic veins can also identify significant TR in prosthetic valves, but care should be taken in interpretation due to potential pre-existing changes unrelated to TV replacement degeneration.

### Pulmonary

The recommendations for evaluating prosthetic pulmonary valves have remained largely unchanged from 2009 (Table 5). They are based on relatively limited data and rely on extrapolated data from the assessment of native pulmonary valves. However, normal values for pulmonary homografts, valved conduits, bioprosthetic and mechanical valves, as well as percutaneous pulmonary valve placed in native outflow tracts are now provided. TTE is the modality of choice for assessing prosthetic pulmonary valve function but may require modified and off-axis views. The main updates to the guidance include the use of CMR and CT in the assessment of prosthetic pulmonary valve and the complementary role they play with TTE.

**Table 5:** Summary of changes for replaced pulmonic valves

Pulmonic valve replacements
**Stenosis** • Measurement parameters used and severity thresholds are unchanged. • Normal values for pulmonary homografts, valved conduits, bioprosthetic and mechanical valves, and percutaneous pulmonary valves placed in native outflow tracts are now provided. • CMR may play a role in assessing the gradient across the PHV/graft. • CCT allows identification of the etiology of dysfunction.
**Regurgitation** • Measurement parameters used and severity thresholds are unchanged. • CMR can be used to quantify regurgitation severity with a regurgitant fraction of >40% suggesting severe prosthetic dysfunction.

Abbreviations: CCT, cardiac computed tomography; CMR, cardiac magnetic resonance imaging; PHV, prosthetic heart valve.

#### Evaluation of Stenosis

As stenosis can occur at any level of prosthetic pulmonary valves, particularly in patients with a valved homograft, the use of pulsed-wave Doppler echocardiography is important to identify the location of obstruction in prosthetic pulmonary stenosis (PS). Quantitative assessment of prosthetic PS focuses on peak velocity and mean gradient, and the thresholds suggestive of significant PS are unchanged from the previous guidance (Table 5). Additionally, the qualitative and indirect measures from serial scans used to determine significant prosthetic PS are also unchanged. CMR may play a role in measuring peak gradient through the pulmonary prosthesis via phase-contrast imaging; however, it is important to note these are often lower than values obtained by Doppler echocardiography. This allows for measuring velocity at different levels to identify the location of obstruction through the prosthesis. The main benefits of CT imaging are in aiding with the identification of the etiology of the stenosis, particularly where artifact prevents accurate assessment by CMR.

#### Evaluation of Regurgitation

As prosthetic pulmonary regurgitation (PR) is relatively uncommon, and due to the scarcity of data, the recommendations for severity assessment are largely unchanged from 2009 and are extrapolated from native PR assessment. Echocardiographic evaluation is generally semiquantitative and includes right ventricular size, color jet width, Doppler intensity, deceleration rate of Doppler, comparison of systemic and pulmonary flows, and diastolic flow reversal in the main pulmonary artery. Echocardiographic assessment is however limited by the presence of eccentric or multiple jets and PVLs and, for TEE, the anterior position of the valve. The main strength of CMR is in the quantification of prosthetic PR via phase contrast imaging, and it may be more accurate than echocardiography in this setting as it is unaffected by eccentric jets or PVLs. It is suggested that a regurgitant fraction of >40% may represent severe prosthetic PR.^2^ Additionally, CMR is the gold standard for assessment of right ventricular volumes, which is important in quantification of PR. CT may have a role in defining the regurgitant orifice area but requires clinical validation.

## Differences With the 2016 European Association of Cardiovascular Imaging Guidelines

Differences between the 2024 ASE guidelines and the 2016 European Association of Cardiovascular Imaging (EACVI) recommendations for assessing PHVs reflect advancements in imaging and management of PHVs over the intervening years.^24^ The 2024 ASE PHV guidelines differ from the 2016 EACVI recommendations in several key areas. The 2024 ASE guidelines incorporate the complimentary use of CMR, CCT, and cardiac PET alongside echocardiography, whereas the 2016 EACVI recommendations primarily focused on echocardiography. The 2024 ASE update also includes new normative values for right-sided PHVs and THVs and updates definitions and diagnostic criteria for bioprosthetic valve dysfunction and PPM. While assessment criteria between the 2 documents are similar, there are minor differences, including the inclusion in the 2016 EACVI criteria for diagnosing PPM, the use of the differences between the reference EOA and measured EOA, and the increase in PHV mean gradient with stress. Both, however, are documents created by expert panels based on years of research and clinical experience and, bearing in mind the 8-year difference in publication date, should be considered complimentary.

## Summary

The 2024 ASE recommendations for PHV assessment with cardiovascular imaging were needed to address advancements in transcatheter heart valves and multimodality imaging. Key changes in this document include recommendations for the complimentary use of CMR, CCT, and PET in addition to echocardiography. Normative values for left-sided PHV have been expanded to include newer prostheses and values for right-sided PHV and THV used in native valves and ViV procedures are now included. Finally, it integrated new definitions to improve the recognition and classification of bioprosthetic heart valve dysfunction and updated diagnostic criteria, including helpful algorithms, for how stenotic PHVs in the aortic position are diagnosed and graded and in the criteria for PPM. These updates have revealed that more information is needed to improve the algorithms and cutoff values for assessing stenotic and regurgitant right-sided PHVs and to validate the use of CMR and CCT methods in these patients. With the ever-expanding use of various transcatheter devices for treatment of both native and PHV dysfunction, future recommendations may incorporate additional guidance on their assessment by cardiovascular imaging.

## Funding

The authors have no funding to report.

## Disclosure Statement

W. Tsang is supported by the Melanie Munk Chair in Advanced Echocardiography. F. Graham has received financial support from Pharmacosmos to attend an international meeting and consultancy fees from Vifor. The other authors had no conflicts to declare.
